# Identification of Subtype-Specific Prognostic Genes for Early-Stage Lung Adenocarcinoma and Squamous Cell Carcinoma Patients Using an Embedded Feature Selection Algorithm

**DOI:** 10.1371/journal.pone.0134630

**Published:** 2015-07-30

**Authors:** Suyan Tian

**Affiliations:** Division of Clinical Epidemiology, The First Hospital of Jilin University, Changchun, Jilin, People’s Republic of China; University of North Carolina School of Medicine, UNITED STATES

## Abstract

The existence of fundamental differences between lung adenocarcinoma (AC) and squamous cell carcinoma (SCC) in their underlying mechanisms motivated us to postulate that specific genes might exist relevant to prognosis of each histology subtype. To test on this research hypothesis, we previously proposed a simple Cox-regression model based feature selection algorithm and identified successfully some subtype-specific prognostic genes when applying this method to real-world data. In this article, we continue our effort on identification of subtype-specific prognostic genes for AC and SCC, and propose a novel embedded feature selection method by extending Threshold Gradient Descent Regularization (TGDR) algorithm and minimizing on a corresponding negative partial likelihood function. Using real-world datasets and simulated ones, we show these two proposed methods have comparable performance whereas the new proposal is superior in terms of model parsimony. Our analysis provides some evidence on the existence of such subtype-specific prognostic genes, more investigation is warranted.

## Introduction

Microarray technology allows simultaneous monitoring of thousands of genes and measuring of their expression values. When data from a microarray experiment being analyzed, a feature selection algorithm, which downsizes the number of genes to a small manageable size, is becoming essential to tackle with difficulties associated with the issue of high dimensionality, namely, the number of genes is much larger than the number of samples. Currently, RNA-sequencing (RNA-seq) has emerged as a novel technology for expression profiles and replaced microarray as the first choice for some biological research, e.g., transcriptomics [1]. Like microarray data, RNA-seq data faces the challenge of high dimensionality. Thus, a feature selection algorithm plays the same crucial role in RNA-seq data analysis as in microarray analysis.

However when we searched on **PubMed** using keywords of *feature selection* and *RNA-seq*, the search only returned 11 articles while the number was 358 after the keyword of RNA-seq being replaced by microarray (dated Jan 12, 2015). The lag of feature selection implementation in RNA-seq data might owe to that RNA-seq data consists of count numbers of sequence reads mapping to each gene. Statistically, count distribution is less tractable than a normal distribution, which is typically used for parametric inference on logarithm-transformed expression measurements in microarray. Recently, a novel function called Voom [2] was proposed, making a normal distribution-based analysis of RNA-seq read count data practically feasible. Voom function has been demonstrated to provide accurate estimation on log Counts-per-million (CPM) values for genes, which we believe can certainly boost the adoption of feature selection algorithms developed for microarray data to RNA-seq data.

Non-small cell lung cancer (NSCLC) is the predominant histological type of lung cancer, accounting for up to 85% of lung cancer cases [3]. The overall five-year survival rate of NSCLC is estimated below 15% because more than two-thirds of NSCLC patients have advanced disease with lymph node and/or visceral metastases at the time of diagnosis [4]. Furthermore, roughly 50% of early stage patients having undergone surgery have and then die of tumor recurrence [5]. Thus precise categorization of early stage NSCLC patients’ prognosis is crucial, and the timely administration of additional therapeutic interventions to the patients with poor prognosis will lead to better survival for them. In contrast, the avoidance of these interventions to patients with good prognosis can reduce medical expenses and improve their quality of life.

Within NSCLC, two major histology subtypes are adenocarcinoma (AC) and squamous cell carcinoma (SCC) with AC approximately accounting for 40% and SCC for 30% of lung cancer cases [6]. Fundamental differences have been found between these two subtypes in the underlying mechanisms of tumor development, growth, and invasion [7,8]. Therefore, successful classification of NSCLC patients into their corresponding subtypes is of clinical importance, e.g. for guiding personalized medicine, and had been previously explored [8–13]. A similarly important question is to consider the prognosis for each histology subtype, and in particular, whether there are subtype-specific biomarkers associated with survival. To the best of our knowledge, studies that consider prognosis can be stratified into the following categories: 1) ignoring histology subtype, e.g.,[5], or 2) focusing on one specific subtype e.g.,[14], or 3) analyzing each subtype separately. None of these means manages to tackle on the identification of subtype-specific prognostic biomarkers directly.

To address this topic specifically, we previously proposed a feature selection method which uses a Cox regression model as a filter to select relevant genes individually [15]. The proposed Cox-model based filter is referred to as Cox-filter herein, and it identifies some subtype-specific genes when being applied to a microarray dataset but fails to do so when applied to a RNA-seq dataset. This might be due to that patients in the microarray study were more homogenous than the RNA-seq data and had been followed up for a substantially longer period. Nevertheless, when including normal paired controls in RNA-seq data, Cox-model filter can also identify some subtype-specific prognostic genes if very liberal cutoffs are chosen.

In this article, we continue our effort on identifying subtype-specific prognostic genes and propose a novel embedded feature selection algorithm. In essence, the method extends Threshold Gradient Descent Regularization (TGDR) algorithm [16] by specifying separate set of parameters for each histology subtype. In TGDR, an objective function is usually defined to be optimized. For instance, log partial likelihood function usually acts as the response function in survival analysis. As an embedded feature selection algorithm, TGDR has its own merits. For instance, TGDR selects relevant feature into the model and estimates its corresponding coefficients simultaneously and thus saves on computing time compared with a wrapped algorithm. Additionally, compared with a filter algorithm TGDR can model dependencies among genes and thus evaluate the coordinated influence of selected genes on the outcome. The use of TGDR in microarray data including survival analysis has been explored previously [9,17–19]. Overall, TGDR has been demonstrated to have superior performance empirically, which encourages us to extend it for the identification of subtype specific prognostic genes. Then using one RNA-seq and one microarray data, we revisit the test on existence of subtype-specific prognostic genes for two major NSCLC histology subtypes. This extension to TGDR is named as Cox-TGDR-specific.

## Materials and Methods

### Experimental data

All data are publicly available from the Gene Expression Omnibus (GEO) repository and The Cancer Genome Atlas (https://tcga-data.nci.nih.gov/tcga/). In those two databases, all personal information on participants is blinded. Both data were previously used by us to illustrate Cox-filter algorithm, our first effort to test existence of NSCLC subtype-specific prognostic genes [15]. Notably based on the statement “… gene expression has the most direct effect on cancer clinical outcomes, and other genome measurements affect outcomes through gene expression. Thus gene expression may carry the richest information on prognosis” by [20], we only consider gene expression profiles for prognosis of AC and SCC in this paper.

RNA-seq data including 489 AC and 488 SCC samples were downloaded from The Cancer Genome Atlas (TCGA), dated Aug 13, 2014. Here, we only considered those patients at early tumor stages, i.e., stage I and II, and adjuvant treatments naïve with clinical outcome (e.g., survival time) accessible. The corresponding data include 70 AC and 55 SCC patients, respectively. Because the event rate (mortality) is very low and both algorithms might lack statistical power to detect significant features in this subset, the restrictions on early-stage and treatment naïve were loosed and extra analysis on those patients at advanced stages was conducted.

Microarray data were retrieved from GEO with an accession number of GSE50081. It was hybridized on Affymetrix HGU133 Plus 2.0 chips. In this cohort, there were 181 early stage NSCLC patients who had not received any adjuvant therapy. Filtering out those samples with ambiguous histologic subtype labels and those other than AC and SCC leads to 127 AC and 42 SCC samples respectively in the final dataset.

### Pre-processing procedures

For the RNA-seq data, Counts-per-million (CPM) values were calculated and log_2_ transformed by Voom function [2] in R limma package [21]. For the microarray data, expression values were obtained using the GCRMA algorithm [22]. Data normalization across samples was carried out using quantile normalization and then expression values were log_2_ transformed.

There are 16,363 unique genes commonly annotated by both datasets. Given the dissimilarity of technology for measuring gene expression values, and the different characteristics of patients in two studies, we performed an integrative correlation coefficient (ICC) [23] analysis to identify genes with consistent co-expression patterns across studies. Downstream analysis was conducted using 7,286 genes passed the ICC filtering with cut-off being set at the median of those ICC values. Then the expression values were further scaled and centralized to have a standard deviation of 1 and a mean of zero.

### Statistical methods

#### Cox-TGDR-specific

As mentioned in **Introduction** section, a response function is indispensable in TGDR. When modelling survival data, a Cox proportional hazards model is usually adopted. Thus its corresponding log partial likelihood function becomes naturally the response function in our proposed Cox-TGDR-specific algorithm.

Without considering the subtype, the survival or censoring time t_ij_, censoring indicator (δ_ij_ = 1 if patient is dead, δ_ij_ = 0 otherwise), and expression values for p genes under consideration X_ij_ = (X_ij1_,…,X_ijp_)^T^ are observed. As is typical for survival data, if δ_ij_ = 1 then t_ij_ corresponds to the survival time, otherwise t_ij_ corresponds to the censoring time. Then the response function is,

$$R\left(\beta \right)=\overset{n}{\underset{i=1}{\sum }}\delta_{i}\left(\beta^{T}X_{i}-log\left(\sum_{k\in r_{i}}exp\left(\beta^{T}X_{i}\right)\right)\right)$$

where **β** is a vector of regression coefficients for genes and r_i_ indexes the set of patients at risk at time t_i_^-^.

To account for the subtype information, a new indicator Y_i_ taking discrete values of c (c = 1,…,C, here C is the number of subtypes) and a separate set of regression coefficients for each subtype **β**_c_ are introduced. Then the corresponding response function is redefined as,

$$R\left(\beta \right)=\overset{n}{\underset{i=1}{\sum }}\delta_{i}\left(\overset{C}{\underset{c=1}{\sum }}\left(I\left(Y_{i}=c\right)\beta_{c}^{T}X_{i}\right)-log\left(\sum_{k\in r_{i}}\overset{C}{\underset{c=1}{\sum }}\left(I\left(Y_{k}=c\right)exp\left(\beta_{c}^{T}X_{k}\right)\right)\right)\right)$$

Here Δ*v* is a small positive increment (e.g., 0.01) in gradient descent search and *v*_*k*_ = k×Δ*v* is the index for the point along the parameter path after *k* steps. Cox-TGDR-specific algorithm is iterated in the following steps,

1. With current estimate β(v_k_), negative gradient matrix *g*(*v*_*k*_) = −∂*R*(*β*)/∂*β* with its (c,j)^th^ component as *g*_*cj*_ *(v*_*k*_ *)* are computed.
2. Let *f*_*c*_(*v*_*k*_) represent the threshold vector of size p for subtype c (c = 1,..,C), its *j* ^*th*^ component is,
  $$f_{cj}\left(v_{k}\right)=I\left(\left|g_{cj}\left(v_{k}\right)\right|\geq \tau_{c}\times \max\left(\left|g_{cl}^{l\in \beta_{c}}\left(v_{k}\right)\right|\right)\right)\;\forall j\in \beta_{c}$$
3. Update β_cj_(v_k+1_) = β_cj_(v_k_)- Δv× g_cj_(v_k_)×f_cj_(v_k_) and *v*_*k*_ by *v*_*k*_+Δv. Step 1–3 is iterated for K times, whose value is determined by cross-validation.

Both K and τs are tuning parameters determining how sparse the final model is. We remark that Cox-TGDR-specific is essentially similar to Multi-TGDR local algorithm [24] only with two differences. First, in Cox-TGDR-specific log partial likelihood function replaces log likelihood function. Optimization on difference response function is because their objective (i.e., Cox-TGDR-specific aims at identifying subtype-specific prognostic genes while Multi-TGDR is at finding genes capable of discriminating multiple subtypes apart) differs. Second, different τ is adopted for each subtype/class in Cox-TGDR-specific. The inclusion of extra tuning parameter certainly increases the computing burden, specifically when determining on their values. Nevertheless, we believe it is somehow a prudent means to address the imbalance of sample sizes/events between two subtypes.

#### Performance Metrics

Using the selected genes, one patient’s risk score was computed. Then, patients were classified into either a low-risk group or a high-risk group based on those risk scores. P-value of a log rank test comparing these two survival profiles was provided. Furthermore, the classification accuracy i.e., the rate of correctly classifying patients into their survival profiles, and the area under Receiver Operating Characteristic (ROC) curve (AUC) statistics were given to assess performance of each algorithm.

### Statistical language and packages

All statistical analysis was carried out in the R language version 3.1 (www.r-project.org), and R codes for Cox-TGDR-specific are available upon request.

## Results and Conclusions

### Real-world applications to NSCLC

**Fig 1:**
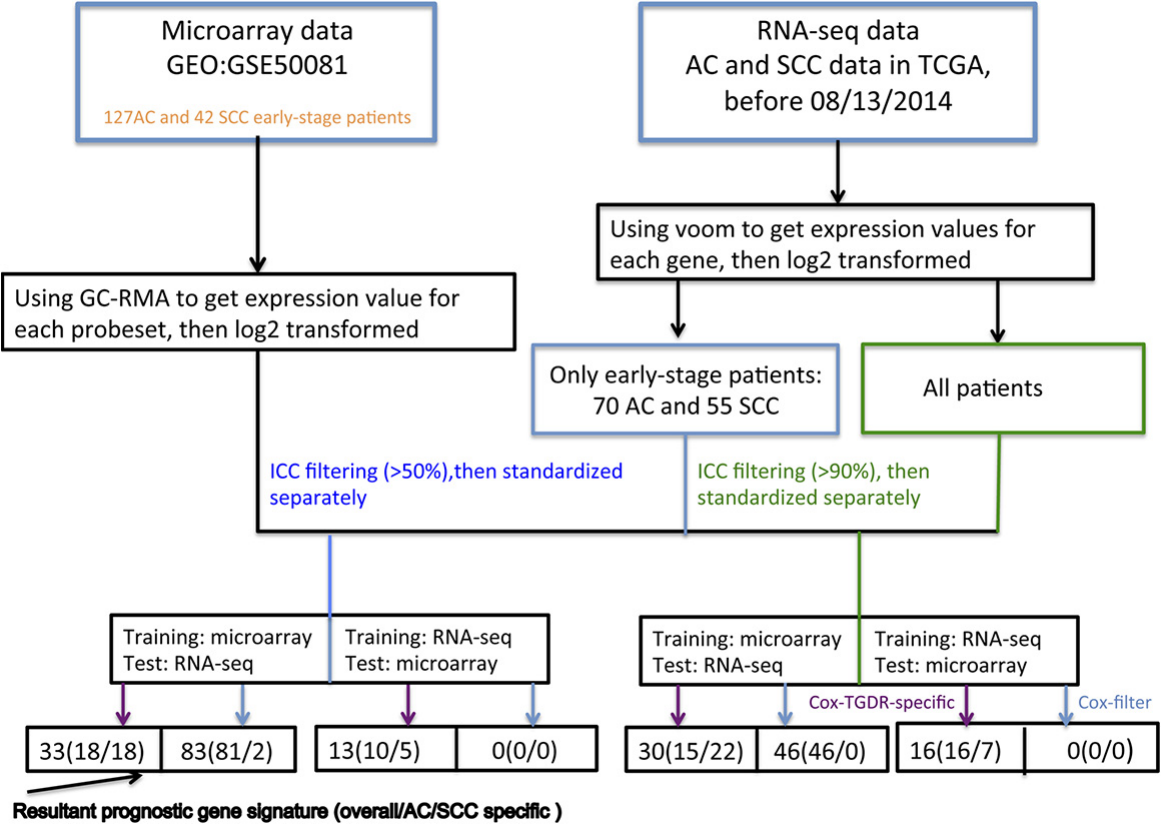
Study schema. A graphical illustration showed how Cox-TGDR-specific and Cox-filter were applied to select relevant subtype-specific prognostic genes for AC and SCC lung cancer.

We applied Cox-TGDR-specific method to both NSCLC datasets. First, we used the microarray dataset as the training set and the RNA-seq data as the test set. Second, we reversed the order and repeated the analysis using the RNA-seq data as the training set, and then tested the resulting markers on the microarray data (Fig 1).

From Table 1A, it was observed that performance of both algorithms was comparable if the training set is the microarray data. When training on the RNA-seq data, only can Cox-TGDR-specific identify some subtype-specific prognostic genes. Moreover, when those resultant signatures were tested on the other dataset from a different platform none of them established any significance.

**Table 1:** Performance of Cox-TGDR-specific and Cox-filter on NSCLC data.

	Cox-TGDR				Cox-filter			
	# genes (# for AC/SCC)	Accuracy (%)	AUC (%) AC/SCC	p-value (log rank)	# genes (# for AC/SCC)	Accuracy (%)	AUC (%) AC/SCC	p-value (log rank)
A. RNA-seq data with patients in early stages (I and II)								
Microarray itself	33 (18/18)	70.4	70.10/86.06	5.55×10^−15^	83(81/2)	76.3	85.23/78.43	1.11×10^−15^
RNA-seq as test		60.8	53.88/67.26	0.154		64	60.34/56.55	0.623
RNA-seq itself	13(10/5)	74.4	55.32/72.32	3.86×10^−3^	0(0/0)	—	—	—
Microarray as test		50.3	51.63/48.32	0.119		—	—	—
B. RNA-seq data with patients in all stages ^1^								
Microarray itself	30(15/22)	65.7	67.21/86.78	3.25×10^−8^	46(46/0)	66.9	77.24/50	0.385
RNA-seq as test		60.9	56.08/52.89	0.167		—	—	—
RNA-seq itself	16(16/7)	72.8	56.85/72.82	2.90×10^−3^	0(0/0)	—	—	—
Microarray as test		51.5	54.57/51.68	0.313		—	—	—

^1^ a higher ICC cut-off (90%) was used.

**Fig 2:**
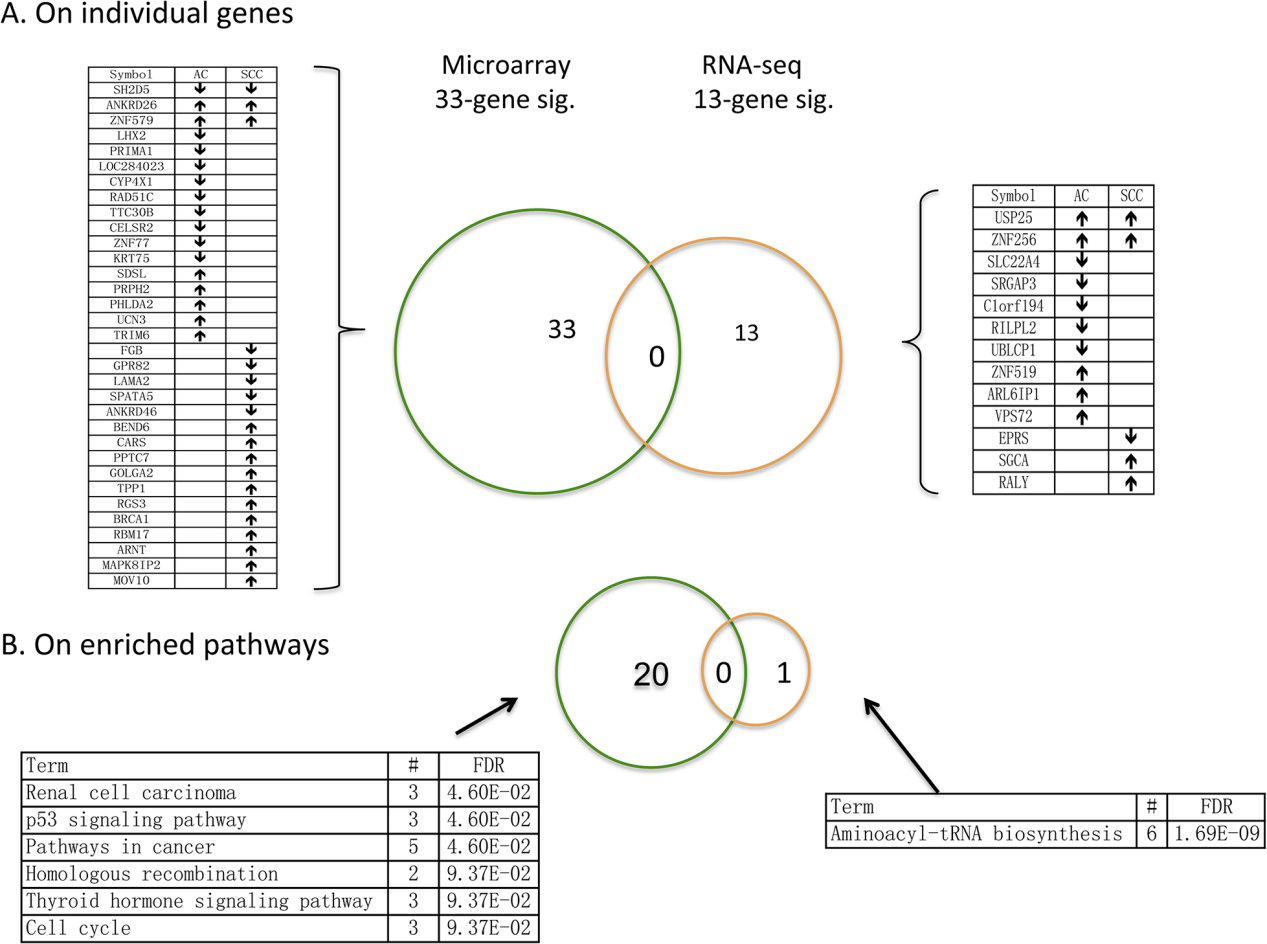
Venn diagrams of 33- and 13-gene signatures. A) On the individual gene level. B) On the enriched pathway level. 33-gene and 13-gene signatures were obtained using Cox-TGDR-specific algorithm with one being trained on the microarray data and the other on the RNA-seq data. Here,↓ and ↑ indicate a negative and positive association with hazard of death, respectively.

Then we examined if the 33 (18 AC/18 SCC specific)-gene signature trained on the microarray data and 13 (10 AC/5 SCC specific)-gene signature trained on the RNA-seq data were consistent and robust. First, we focused on the individual gene level. Afterwards, we shifted our attention to the pathway level and evaluated how the enriched pathways by these two signatures overlap (Fig 2). The search of enriched pathways was conducted using a web-based database called STRING [25]. No overlap between these two signatures on both levels and successful identification of *keratin 5* (KRT5) as a discriminative gene between AC and SCC samples (although training on data from different platforms) [13] partially justify the claim that prognosis prediction using gene expression profiles is a more difficult task than membership/class prediction [20,26].

Even though our goal is to develop histology subtype-specific prognostic genes for early-stage AC and SCC, the RNA-seq data including only early-stage patients has major drawbacks, i.e., small samples and few events (19 death among 125 patients). These might explain why Cox-filter cannot identify any significant genes. Thus, we released the restriction on stages and applied both Cox-filter and Cox-TGDR-specific algorithms to the whole RNA-seq dataset. Notably, using the whole RNA-seq set leads to more heterogeneity between microarray data and RNA-seq data. To alleviate this, we set ICC filtering threshold at a higher value, i.e., 90%. The results are presented in Table 1B.

Unfortunately, no meaningful results have been obtained. To explore if different cut-off on ICC filtering influences the results, we conducted a sensitivity analysis by varying its value over a grid of 0.5~0.9 with an increment of 0.1, and found out the conclusion is consistently negative across those values. Furthermore, given the event (death) rate is still as low as around 30%, we randomly excluded 50% of those censored patients and applied both Cox-filter and Cox-TGDR-specific algorithms to the combined data of the remaining 50% censored patients and those decreased. Again, the conclusions are consistent (Data not shown). We attribute the invalidity of resultant signatures’ being applied to independent data to heterogeneity of study populations, platforms, protocols and so on.

One may argue that the resultant prognostic signatures should contain many robust differentially expressed genes (DEGs) between AC and SCC. After an examination on those markers, we think it is unsurprising to have two disjointed sets for prognostic signature and DEGs. First, the objective here is to find subtype-specific prognostic genes. Therefore, the outcome is survival time versus that is gene expression value in typical DEG identification setting. Those DEGs between AC and SCC might not be associated with their respective survival rate. For instant, none of *tumor protein p63* (TP63) and *NK2 homeobox 1*(NKX2-1) was identified by us, or by the 13-gene signature in Guo et.al [28] and the 15-gene signature in Zhu et.al [29]. Those two studies are highly relevant to our study, with the objective of finding prognostic genes for early-stage AC and SCC lung cancer.

Furthermore, an embedded feature selection method tends to be superior in terms of model parsimony. It means an embedded method is capable of selecting few features among those highly correlated ones. For example, in one extreme case only KRT5 [10] was identified as relevant for telling AC and SCC samples apart while other DEGs were considered as irrelevant. Nevertheless, two pathways–renal cell carcinoma and pathways in cancer to which *Kirsten rat sarcoma viral oncogene homolog* (KRAS) and *phosphatidylinositol 3-kinase* (PI3K) belong are among enriched pathways by the 33-gene prognostic signature trained on the microarray data.

Lastly, in order to obtain a robust prognostic gene signature across different platforms, we only considered those genes that present consistent expression pattern between the microarray and RNA-seq data in this study. As a result, some of those DEGs such as *fibroblast growth factor receptor 1* (FGFR1) and PI3K were filtered out.

### Synthesized data

To explore the characteristics of Cox-TGDR-specific and compare it with Cox-filter algorithm, we conducted simulations using gene expression values of the microarray data. Specifically, we chose four genes–CERCAM, ITGA5, MTHFD1L, and PLOD1 –to be prognostic markers, and then randomly selected 96 genes to make the total number of considered features as 100. Two extreme cases were explored: 1) Mutually exclusive markers for each subtype where genes 1 and 2 are associated with SCC versus genes 3 and 4 with AC, and 2) no subtype specific prognostic genes where all genes 1–4 share a common hazard function for both subtypes.

Notably, we actually duplicated the simulations we set up previously except two minor differences. One is here expression values of each gene were standardized and centralized because such extra scaling is required in Cox-TGDR-specific to avoid the algorithm being dominated by genes with big variability, the other is the number of replicates is 50 because Cox-TGDR-specific is so computing intensive that 500 replicates will consume hours to be finished, and 50 replicates are fairly adequate to identify meaningful patterns. For more details on the simulations, our previous work [15] is referred.

Table 2 summarizes the simulation results. In both cases, we observed that Cox-TGDR-specific outperformed Cox-filter in terms of parsimony. As a result, the false true rate is low for Cox-TGDR-specific. This is especially true in the case of no subtype specific genes. Also in this case, however, Cox-TGDR-specific fails to identify gene 4, implying its false negative rate might be higher. Given the signal of this gene is weaker compared to other three genes, the failure of Cox-TGDR-specific to identify it might not be a big surprise. Similarly, one possible explanation for both algorithms’ failure of identifying gene 2 as a SCC-specific prognostic gene is its signal is substantially weaker compared to gene 1.

**Table 2:** Performance of Cox-TGDR-specific and Cox-filter on simulated data.

	Cox-filter		Cox-TGDR-specific	
	AC (%)^1^	SCC (%)^1^	AC (%)^1^	SCC (%)^1^
A. Simulation 1: mutually exclusive markers for each subtype				
Gene1	0	100	100	98
Gene2	0	0	0	0
Gene3	100	0	100	14
Gene4	100	0	100	26
No. of selected genes	9.76	7.72	3.4	3.82
B. Simulation 2: no subtype specific prognostic genes				
Gene1	100	100	100	100
Gene2	100	100	100	100
Gene3	100	100	100	98
Gene4	100	100	0	0
No. of selected genes	24.48	31.33	3.54	4.14

^1^ represents the percentage with a non-zero coefficient for specific gene among 50 replicates.

## Discussion

In this article, we introduce an embedded feature selection algorithm called Cox-TGDR-specific. Similar to Cox-filter algorithm, it owns the capacity of selecting subtype-specific prognostic genes. Nevertheless, different from Cox-filter it can select relevant genes and estimate magnitudes of those genes’ association with outcome simultaneously. Certainly, it comes at an extra computing cost given in Cox-filter feature selection is realized using a simple Cox-model and coefficient estimation is separated from this step. Furthermore, cross-validations are needed in order to find the optimal tuning parameters in Cox-TGDR-specific, which adds computing burden. Nevertheless, although Cox-filter is apparently free of tuning parameters a sensitivity analysis is usually in demand to decide on an optimal cutoff for FDR, which varies from one real-world application to another.

As shown by simulations and one real-world application, Cox-TGDR-specific outperforms Cox-filter in terms of model parsimony. Also because we use different tuning parameter τ for each subtype, the imbalance of sample sizes between two subtypes can be taken into account. This point is justified by the fact that compared to Cox-filter, the performance statistics of SCC-specific genes are dominantly superior in Cox-TGDR-specific. Certainly, different cutoff values for FDR in Cox-filter can be adopted to improve upon the performance of Cox-filter. Since it is beyond the scope of this article, this topic is skipped.

Different from Cox-filter that is an analysis-of-marginal-effects method, Cox-TGDR-specific is an analysis-of-joint-effects method [27]. Even though an analysis-of-joint-effects method simultaneously accounts for the effects of all selected genes, Cox-TGDR-specific does not perform excellently and superiorly as we expect. By grouping those genes presenting co-expression patterns into separate subsets and applying regularization firstly to those groups and then to individual genes within the selected groups hierarchically, Ma et al. [17] demonstrated their proposed algorithm is superior to the regular TGDR on survival data. Therefore, to develop an algorithm to incorporate co-expression pattern/biological relevancy as a priori, so that it can identify subtype-specific prognostic genes and achieve more accurate prediction on a patient’s prognosis, is definitely our future research.
